# Portable Facial Expression System Based on EMG Sensors and Machine Learning Models

**DOI:** 10.3390/s24113350

**Published:** 2024-05-23

**Authors:** Paola A. Sanipatín-Díaz, Paul D. Rosero-Montalvo, Wilmar Hernandez

**Affiliations:** 1 SDAS Research Group, Hay Moulay Rachid, Ben Guerir 43150, Morocco; paola.sanipatin@sdas-group.com; 2 Computer Science Department, IT University of Copenhagen, 2300 Copenhagen, Denmark; paur@itu.dk; 3 Carrera de Ingenieria Electronica y Automatizacion, Facultad de Ingenieria y Ciencias Aplicadas, Universidad de Las Americas, Quito 170124, Ecuador

**Keywords:** electromyography sensors, facial expressions, machine learning

## Abstract

One of the biggest challenges of computers is collecting data from human behavior, such as interpreting human emotions. Traditionally, this process is carried out by computer vision or multichannel electroencephalograms. However, they comprise heavy computational resources, far from final users or where the dataset was made. On the other side, sensors can capture muscle reactions and respond on the spot, preserving information locally without using robust computers. Therefore, the research subject is the recognition of the six primary human emotions using electromyography sensors in a portable device. They are placed on specific facial muscles to detect happiness, anger, surprise, fear, sadness, and disgust. The experimental results showed that when working with the CortexM0 microcontroller, enough computational capabilities were achieved to store a deep learning model with a classification store of 92%. Furthermore, we demonstrate the necessity of collecting data from natural environments and how they need to be processed by a machine learning pipeline.

## 1. Introduction

In recent years, humans and machines have worked closely to understand the human brain and its functionalities to control body movements [1,2]. Specifically, machines try to recognize human behavior through muscle activity to interact with them [3,4]. Therefore, facial muscles play a fundamental role in human communication, even with machines, since facial expressions represent a very high percentage of nonverbal communication, where 55% of the message is transmitted through facial expressions, 38% by intonation, and only 7% of the message by linguistic language [5]. Hence, smooth communication between people and machines requires interfaces, which commonly are sensors or cameras. However, cameras use remote sensing techniques to collect data from humans whereby, in some scenarios, these techniques cannot describe muscle movements when people react to a specific emotion [6]. Indeed, cameras can recognize emotions by applying deep learning techniques comparing thousands of images. These techniques require heavy computational resources, mainly when the deep model applies filters to find patterns that remove unnecessary picture information. Furthermore, when cameras are deployed in uncontrolled environments, they insert luminosity variations and vibrations that affect the quality of the image and the ability of the machine to recognize human emotions. As a result, cameras struggle to detect the voluntary/involuntary messages produced by the nervous system in response to human emotions [7].

Conversely, modern data acquisition systems collect data from human behavior through electrical measurements of muscle activity. Furthermore, emotion recognition methods (ERMs) attempt to recognize facial expressions [8,9] in different situations to determine the internal emotional state of a person by subjective experiences (i.e., how the person feels) [10]. ERM techniques are focused on gathering information from physiological signals, such as electroencephalogram (EEG), electromyogram (EMG), and electrocardiogram (ECG) [11]. Previous work, such as Ekman [12], proposes the existence of six basic facial emotions: anger, disgust, fear, happiness, sadness, and surprise. These emotions follow a specific physiological response pattern and can be demonstrated by ERM techniques, such as EMG analysis. Therefore, collecting EMG signals need small electrodes (i.e., sensors) to detect light voltage variations in the muscles of the face [13]. In addition, it is important to mention that facial muscles have a single motor unit associated with movements of different facial regions which contribute to the representation of facial expressions and that is located under the subcutaneous tissue of the face and neck [14].

Taking into account the abovementioned statements, the research goal is to present an EMG electronic device that can classify the most relevant human emotions locally [15]. Therefore, the system uses three sensors placed on specific muscles of the face to collect data and create a dataset. Then, filters in hardware and software are developed to reduce noise and unpredictable light muscle contractions of the face. Next, three machine learning (ML) approaches are designed to define a suitable ML method that has the highest classification accuracy and low computational consumption. In short, the main contributions are as follows:

- An extensive literature review is carried out to select the minimum number of sensors and samples to classify six human emotions, proving that EMG analysis is an adequate alternative in harsh environments where cameras struggle to take high-quality images of humans.
- A proper ML analysis is performed using three approaches to determine the best one with a light workload in the electronic device. Therefore, analog signals are converted into different data structures to fit ML algorithms’ training phase.
- An adequate electronic system design is presented, which combines hardware and software to reach an ML application, keeping a high classification score and less power consumption than cameras.

The rest of the manuscript is structured as follows: Section 2 shows related works and a background of facial muscles. Then, Section 3 presents the electronic design focused on the location of sensors. Afterwards, Section 4 describes the machine learning pipeline. Next, the results are discussed in Section 5. Finally, Section 6 presents the conclusions of the paper and future work.

## 2. Background

This section summarizes earlier surveys covering the intersection between the data acquisition and deep learning techniques used to achieve emotion recognition (Section 2.1). Following this, Section 2.2 shows a facial muscle description for a better understanding of the location of sensors.

### 2.1. Early Works on EMG in the Field of Emotion Recognition

In detection-based computer vision, Thiam et al. [16] developed their own data classification pipeline, including three cameras to acquire a human emotion database, with results that yielded an accuracy of about 94%. In addition, Perusquia et al. [17] introduced a device for micro-smile recognition using signal processing and neural networks. These works demonstrate that cameras need controlled environments and an external server to deploy deep learning models. On the other hand, early studies such as [11,18,19,20,21] present emotion recognition approaches with classical classifiers, such as support vector machines (SVMs) and convolutional neural networks (CNNs). They mainly use ECG and EMG signals. Furthermore, recent works such as [22,23,24] have presented extensive methods to classify emotion recognition by means of EEG signals, achieving 97% accuracy. However, they use existing datasets to develop their neural network models [25]. Finally, Pham et al. [8] presented a fine-tuned CNN model focused on emotion recognition.

In EMG signals space, Jiang et al. [26] presented one of the first intuitions to recognize emotions trough EMG signals. Then, works such as [13,27,28] presented electronic devices to acquire EMG signals from some facial muscles. The studies included SVM and neural networks, with an accuracy of about 91% up to 99% by taking thousand of samples and running experiments on computers. Therefore, the abovementioned electronic devices are developed just for data collection. Furthermore, in [29], the authors presented a multichannel EMG analysis and a long short-term memory classifier (LSTM) as a classifier to recognize eight emotions with 95% average accuracy. Moreover, another study on emotion recognition, which also focused on the recognition of facial expressions using EMG signals, is presented in [30].

The abovementioned works have complex systems, and their application in natural conditions would be challenging since they depend on the number of sensors and human emotions collected. In addition, they might need high computational resources and specific hardware in controlled environments. Therefore, few applications have worked on exporting the ML model to the device, which offers proof that its portability is not high.

Moreover, other previous works include [31,32,33]. In [31], a method for reading human facial expressions using a wearable device is presented. The method combines independent component analysis (ICA) and an artificial neural network (ANN). In addition, the authors are able to identify smiling and nonsmiling faces by measuring bioelectrical signals on the side of the face. However, EMG information from the corrugator supercilii and zygomaticis major muscles was not recorded. Similarly, ref. [32] used a wearable, noninvasive method to study facial muscle activation associated with masked smiles, enjoyment smiles, and social smiles. The method presented in [32] uses EMG recording, ICA, and a short training procedure. However, according to [33], ref. [32] does not show the degree of agreement (i.e., concordance) between facial EMG data and subjective valence.

On the other hand, in contrast to this [31,32], ref. [33] provide evidence that emotional valence can be assessed using a wearable facial EMG device while we are moving, The wearable device developed in [33] recorded the corrugator supercilii and zygomatic major muscles.

Finally, the wearable devices mentioned above are electronic systems placed on the head, near the cerebral cortex. Therefore, from our point of view, it would be useful to study the emotions that people experience when using battery-powered electronic devices near the cerebral cortex, and also how these devices affect people’s health. In the work presented in this research, we try to distance the electrical part of our measurement system from the user. In this way, we avoid any kind of interference with the functioning of the brain while we classify people’s expressions. We also use both hardware and software filtering to reduce the noise and complexity of the ML model. Lastly, the models presented in this work can be translated into portable electronic systems with low computational resources and low cost.

### 2.2. Facial Muscles

The facial muscles are the fibers (elastic tissue) that support sensory organs to perform their activities, for example, chewing, smelling, hearing, or listening. Motor neurons carry out this process by giving instructions to muscles by electrical pulses to maintain muscle contraction. This electrical activity is called motor unit action potential (MUAP), representing the duration, electrical amplitude, and phase of muscle contraction. Typical MUAP duration is between 5 and 15 ms [34]. In this scenario, the facial muscles are divided into three groups: (1) the top group, which generates eyebrow movement, frowning mainly to allow eye-opening; (2) the middle group, which is closely involved with mouth movements and helps phonation, and some very particular muscles allow chewing; and (3) the bottom group, which is responsible for generating facial movements due to the lips’ soft tissues [35].

The recognition of facial expressions from physiological signals is generated from the central nervous system through the MUAP analysis. Thus, there are low electrical levels when the muscle is at rest. In contrast, muscle contraction is generated when a nerve impulse raises the electrical levels [36]. Therefore, the facial actions coding system (FACS) indexes the facial expressions in actions units (AUs) to match the biomechanic information obtained in MUAP analysis of EMG signals. Furthermore, this approach aims at splitting the face into individual movements of their specific muscle activation to describe emotions [37,38]. Therefore, FACS has intensity scoring by appending letters A–E (for minimal–maximal intensity) to the action unit number. Consequently, the target emotions that FACS helps to determine are as follows: surprise, sadness, happiness, anger, disgust, and fear. Lastly, Table 1 shows the summary of all the muscles people commonly use to express emotions according to FACS.

## 3. Electronic Design

Section 3.1 mentions setting up sensors for the data collection process. Then, Section 3.2 describes the electronic system.

### 3.1. Sensors’ Location

EMG sensors are composed of two units. The first unit consists of electrodes to collect electricity fluctuations in muscles. EMG sensors are usually built with three electrodes: two electrodes are placed on the top and in the middle of the muscle, and the last one is considered ground, commonly placed on bones since they do not have electricity [39]. The second unit is in charge of cleaning incoming data from electrodes; it has an instrumentation amplifier internally with a gain of 200×, a bandpass filter with a lower 3 dB cutoff frequency of 20 Hz and an upper 3 dB cutoff frequency of 498 Hz, and a rectification circuit [40].

**Figure 1:**
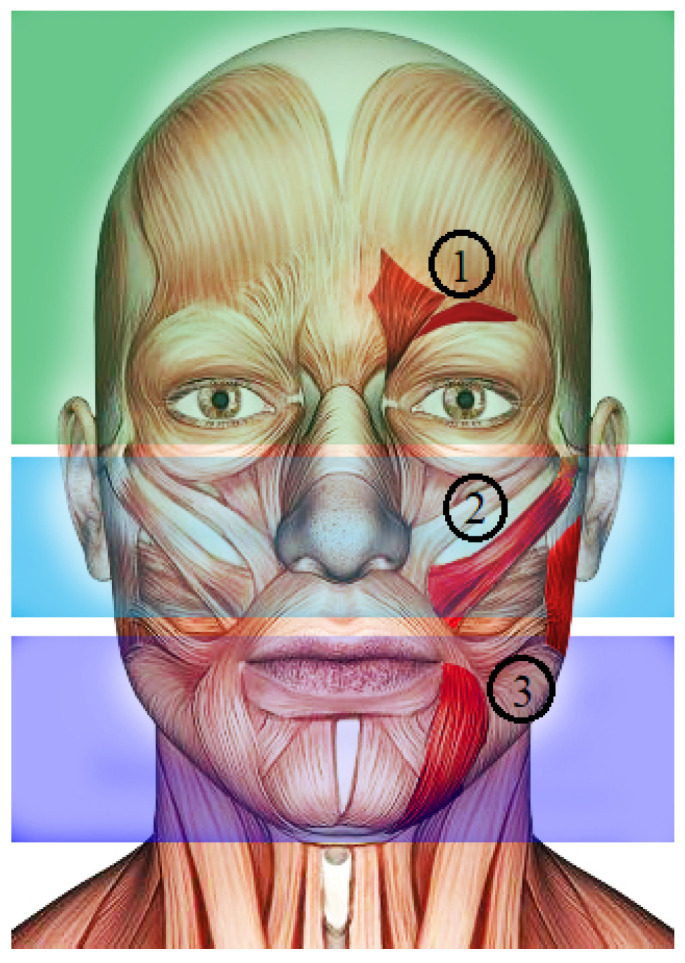
Location of three EMG sensors to collect data. Sensor 1: Orbicularis oculi and the Corrugator supercilii muscles; Sensor 2: Zygomaticus major; Sensor 3: Depressor Anguli Oris and Masseter muscles. Green section: top face muscles; Blue section: middle face muscles; Purple section: bottom face muscles.

Following the EMG sensors configuration, Table 1 shows the primary muscles activated in the target facial expressions. We figured out that some muscles are part of several expressions. Therefore, we determined three EMGs in the most participating muscles to describe each facial expression. One sensor was placed between the *Orbicularis oculi* muscle and the *Corrugator supercilii* muscle. These muscles are in the forehead’s upper part of the eyebrow. It is the primary muscle over the negative emotions of sadness, anger, and disgust (sensor 1). Another sensor was placed on the *Zygomaticus major* muscle, which is located in the cheek near the mouth. This muscle highly acts on emotions of happiness, fear, and surprise (sensor 2). Finally, the last sensor collects data from the Depressor Anguli Oris and Masseter muscles. Both muscles are in the lower part of the mouth, towards the jaw, and act mainly on the emotions of surprise, fear, happiness, and sadness (sensor 3) [37]. With this sensor selection, we ensure that at least two sensors are activated on each facial expression. In short, Figure 1 shows the graphical description for the sensors’ locations (Figure 1 was purchased by the authors in https://sp.depositphotos.com/41323531/stock-photo-male-anatomy-face.html (accessed on 21 November 2023) depositphotos and modified by them to adapt it to the interests of the proposed research).

### 3.2. Electronic System Description

**Figure 2:**
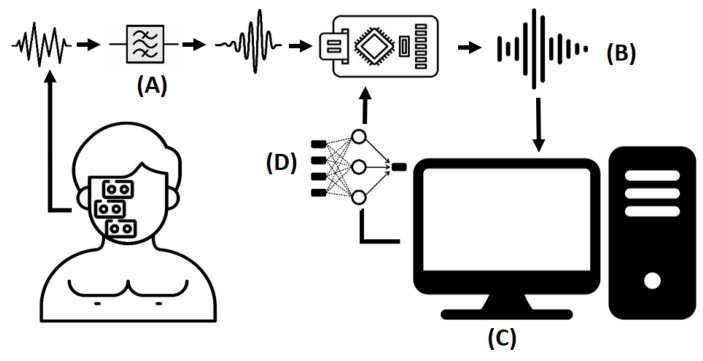
Electronic system design. Step (A): Sensors gather data and analog filtering is carried out. Step (B): The microcontroller receives the EMG signals and converts them into digital signals to send to the computer. Step (C): The computer stores the EMG signals to train ML models. Step (D): The inference is allocated to the microcontroller.

Once each sensor’s location is defined, the next step is designing the portable electronic device. According to the hardware’s functionalities and replicability requirements, MyoWare muscle biosensors were selected to collect EMG signals. These sensors are placed in a bipolar configuration with at least 2 cm of separation between them to avoid noise caused by electrical interference. Then, to avoid the sensor sending a raw signal directly to the microcontroller, an extra operational amplifier is used to couple the signal to the analog–digital converter and reduce noise for unpredictable facial movements. This task is carried out by using a voltage follower amplifier. However, even when the signal is rectified, some DC components, represented as signal peaks, are part of the EMG signal. Therefore, signal filtering is needed in further steps. The Arduino nano IoT was defined as a microcontroller to process EMG signals. Next, the Arduino sent those signals to the computer to store them and train ML models. Finally, once the ML model was selected, it was optimized to run into the microcontroller and make decisions locally. Figure 2 shows the abovementioned electronic system design.

## 4. Machine Learning Pipeline

Once the electronic prototype is described above, it can collect EMG data from people. However, even when data play a fundamental role in discovering facial expression patterns, it is necessary to establish an adequate ML pipeline to select ML models that fit the data and test them with the proper performance metrics. In this scenario, we defined the ML pipeline with the following steps: data collection, data preprocessing, data preparation, and model design. Moreover, the following section describes how to evaluate the model, optimize it, and export it to the electronic device.

### 4.1. Data Collection

**Figure 3:**
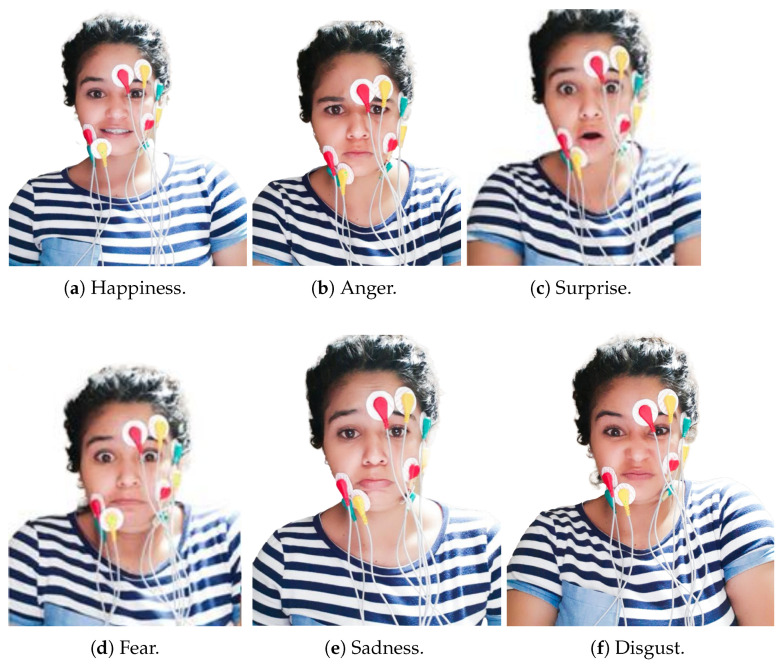
Samples reshaped into one-dimensional array.

For conducting the data collection stage, 30 people (i.e., 18 men and 12 women) used cleaning/shaving products to remove beards and make-up. Then, each person used new electrodes to collect data. The central frequency was 25 Hz, and the system took 300 samples around it in 4 s, where the first and the last second usually contained errors due to a person’s delayed response to activate their muscles. Then, people rested for 4 s between experiments to eliminate the effect of muscle fatigue [41]. This process was performed ten times, where people intentionally made each emotion by their criteria, representing 300 instances per expression. Afterward, they were exposed to images to express emotions naturally under the same experimental conditions. We used a Lenovo Thinkpad P50s laptop system to store data; its specifications are listed in Table 2. Figure 3 shows the sensors’ locations and the six facial expressions taken into consideration in this research.

### 4.2. Data Preprocessing

**Figure 4:**
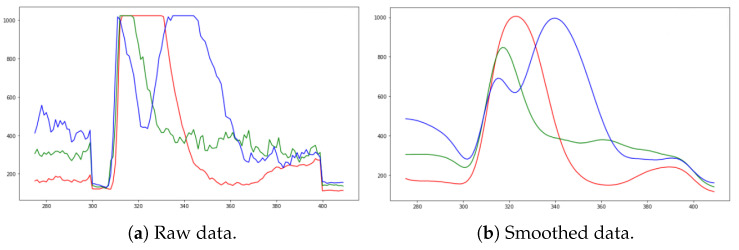
Anger representation by EMG signals. Y-axis: Analog-to-digital converter resolution; X-axis: Sample size. Sensor 1: (–); Sensor 2: (–); Sensor 3: (–).

The dataset was built once EMG signals were collected and stored in the computer. The dataset shows the six types of facial expressions: happiness, anger, surprise, fear, disgust, and sadness. Each sample is a three-dimensional array (i.e., 100 instances per expression with 3 EMG sensors [3][100]), representing 3600 samples with their respective labels. However, when these samples are plotted, it is evident that some noise components were injected into the analog signal. Therefore, smoothing filters in software were deployed to improve the signal. The signal-to-noise (SNR) metric determines the suitable smooth algorithm [42,43,44]. The results are shown in Table 3. In addition, this table shows a statistical description of the signal: mean, standard deviation (SD), and variability coefficient. This statistical information provides the proper smoothing filter, which is the Gaussian filter, since it has less variability in the smoothed samples and better SNR. Figure 4 shows an example of how the Gaussian filter smooths the signal and reduces the noise.

### 4.3. Data Preparation

**Figure 5:**
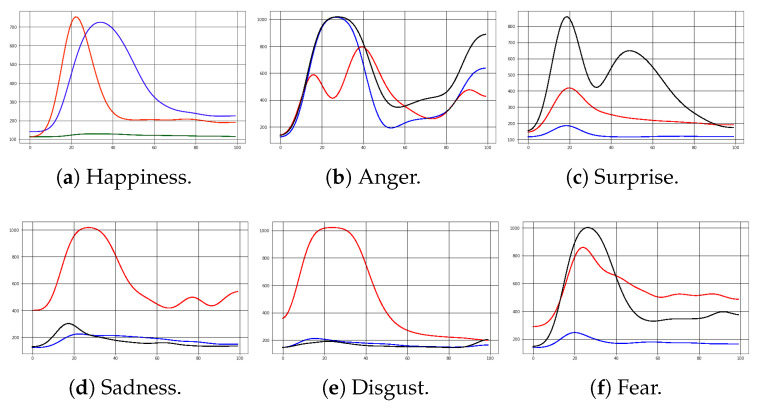
Multidimensional EMG signals. Y-axis: Analog-to-digital converter resolution; X-axis: Sample size. Sensor 1: (–); Sensor 2: (–); Sensor 3: (–) or (–).

**Figure 6:**
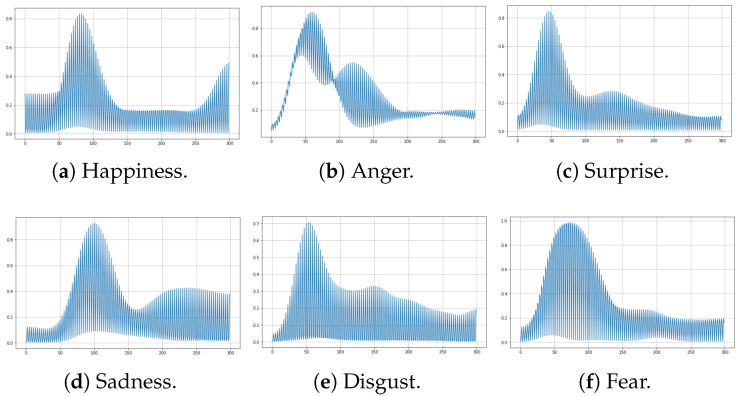
One-dimensional EMG signal. Y-axis: Analog-to-digital converter resolution; X-axis: Sample size.

Once the EMG data are clean, data must be reshaped to fit for ML models. First, deep learning methods allow multidimensional data as model inputs, in compliance with how the dataset is already presented (i.e., each sample is an [100][3] array). However, classical ML methods described in further sections require unidimensional data as model input. Therefore, the dataset was reshaped into one-dimensional data by merging three sensors into one signal. This was achieved by adding the last two sensors to the end of the first sensor. As a result, each sample changed to a [300][1] array. Both datasets were split across participants into the training and test set, taking 80% and 20%, respectively. This is performed to obtain generalized models and obtain unbiased results. This procedure was carried out ten times by taking random samples each time to obtain balanced classification results. Figure 5 and Figure 6 show samples of multidimensional and one-dimensional data of each facial expression.

### 4.4. Model Design

This subsection shows the supervised classification algorithms with the one-dimensional training set and the deep learning algorithms with the multidimensional training set.

#### 4.4.1. Supervised Classification Algorithms

Classification algorithms can learn using different mathematical approaches. In consequence, reviewed works (e.g., [24,45,46]) mention the most usual classification algorithm methods based on the following:

- Distance-based: K-nearest neighbors (KNN).
- Model-based: Support vector machine (SVM).
- Density-based: Bayesian classifier (BC).
- Heuristic: Decision tree (DT).

#### 4.4.2. Neural Networks

Neural networks learn to extract relevant features when data pass through their architecture, and each neuron updates its weight to create probability rules. Therefore, they can learn from time series, sound signals, and images. Given that the dataset is presented in two ways (i.e., multidimensional and reshaped one-dimensional input), two neural network models were deployed with similar structures but different input shapes. The first model is shown in Table 4, called DP_1_. The DP_1_ architecture uses a sequential model and dense layers, which means that each layer is deeply connected with its preceding layer. In addition, “rectifier linear unit” (ReLU) is used for sparse activation of neurons, efficient computation, and better gradient propagation. Then, a flatten layer is used to obtain a flattened output. Finally, a dense layer with six neurons with the softmax activation function is added. Applying softmax makes the output vector in the interval I=[0,1] such that the components will add up to 1. Therefore, they can be interpreted as probabilities. In this research, we do not use CNN architectures because in previous tests we could not achieve the high accuracy of standard neural networks, even when we tried several architectures.

Consequently, the second neural network model, DP_2_, uses a similar architecture to the first model except for the flatten layer since the input is a one-dimensional array (see Table 5). It is worth mentioning that both neural network models do not add a drop layer, because we obtained this architecture based on trial-and-error experiments to avoid overfitting and reduce the model complexity.

## 5. Results

This section shows the evaluation of the ML models using classification metrics provided by the confusion matrix. Then, the selected ML models are optimized to be placed on the microcontroller’s memory and make inferences locally. Finally, the electronic design is presented.

### 5.1. Evaluation of ML Models

The confusion matrix tested the ML models’ performance with the test set. It provides the following classification metrics: precision, recall, F1-score, and accuracy. Table 6 shows that KNN and SVM algorithms are unsuitable for recognizing facial expressions since their performance is lower than expected. Furthermore, both techniques struggled to identify surprise and disgust. Conversely, naive Bayes and decision tree have high scores in classifying facial expressions even when the precision in detecting disgust is below 90%. Related to deep learning models, the DP_2_ model had a similar performance to classification algorithms (i.e., accuracy is about 93%). On the other hand, DP_1_ proved to be the model with the highest classification accuracy, recall, and precision. Consequently, the decision tree algorithm and the neural network models DP_1_ and DP_2_ are quantized and exported into the microcontroller’s memory to test them in a real-world environment to predict the label in incoming data (i.e., make inferences locally). Naive Bayes is not considered for further stages since it does not have support to quantize the model and needs to be trained on the device, which demands high computational requirements.

### 5.2. Model Optimization and Deployment

A real-world test was designed to collect data from five women and ten men who were not considered when the ML models were trained. The testing methodology started when participants expressed different facial expressions without external stimuli. Afterward, the participant was exposed to see images and reacted to them naturally. First, the decision tree algorithm was placed on the microcontroller’s memory to predict the facial expression of people. This model needs 4 Kbytes of flash and 2 Kbytes of RAM to make inferences in 2 s. The model’s accuracy when external factors do not stimulate people is 79%, and when they are stimulated, it is 85%. Second, the DP_1_ model was also placed on the microcontroller’s memory to run the same test; this model uses 150 Kbytes of flash and 10 Mbytes of RAM and makes decisions in 8 s. This model could recognize facial expressions at 88% and 92%, respectively. For its part, DP_2_ uses 120 Kbytes of flash and 6.5 Mbytes of RAM and makes decisions in 5.5 s. The DP_2_ recognizes facial expression at 86% and 90%, respectively. Therefore, this model was selected to design the final device since, even when its performance is lower than DP_2_, it consumes 55 mW rather than 80 mW, which is a large improvement in the power consumption while keeping a similar performance.

### 5.3. Electronic Device

**Figure 7:**
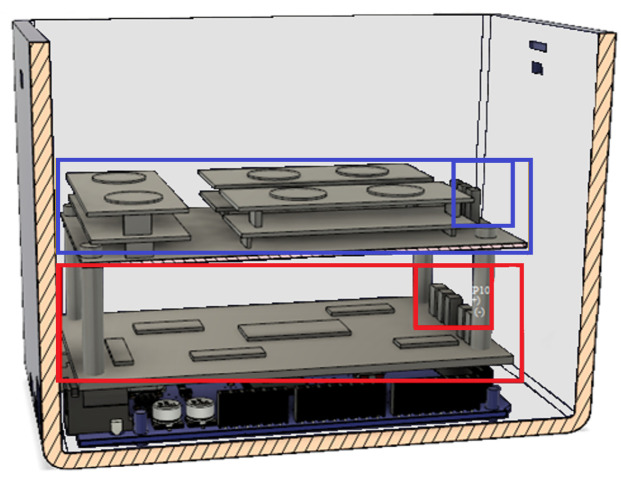
Digital electronic system. Blue rectangle: sensors’ board; small blue and red boxes: jumpers; red rectangle: noise filter board placed allocated over the microcontroller.

After selecting the ML model, the next step was to design the PCB board and the device’s case. We aimed to build a robust electronic board and designed two electronic shields that would connect with the carrier board male headers. The first shield was placed on top of the carrier board and consisted of analog amplifiers to couple the EMG signals with the analog–digital pins of the microcontroller. The second shield was for the sensors and was placed on top of the first shield. Both shields were powered by an external supply and connected by jumpers. The system can be seen in Figure 7.

**Figure 8:**
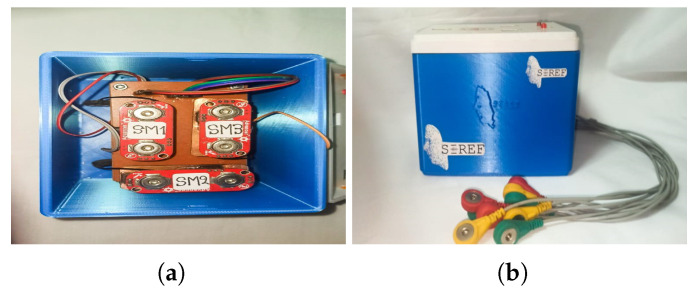
Emotion recognition electronic case. (**a**) Sensors’ PCB board. (**b**) Electronic case with electrodes.

In addition, Figure 8 shows the electronic case made in a 3D printer. On top of the case, there are LEDs to highlight when the electronic system collects data and makes decisions. A USB connection is also supported to power the system, recharge the battery, and send data to the computer.

## 6. Conclusions and Future Work

In this paper, the proposed facial expression recognition system by EMG sensors and ML methods, which integrates an ML pipeline and electronic system design, performed excellently in classifying the six principal facial expressions. In addition, a deep learning model with a multidimensional array as input was shown to be an acceptable solution compared to classification methods. However, when multidimensional data were reshaped to one-dimensional data, it achieves similar performance but with lower power consumption. This power consumption is an adequate improvement in electronic devices that need batteries. Additionally, the deep learning model was quantized to be compiled into the microcontroller to reduce sending information to the cloud. Therefore, the electronic system is portable and can send information to computers with low computational resources since the model does not need to be updated. Furthermore, the ML pipeline shows an adequate data collection step with the correct allocation of EMG sensors on the face using the facial actions coding system, which describes the specific facial muscle activation. As a result, the electronic system was built with a case with a noise cancellation board between the sensors and the microcontroller.

Moreover, the deep learning model can correctly recognize over 90% of human expressions in women and men. In addition, it demonstrated that artificial expressions without stimuli could be detected with sensors and showed that natural facial expressions are standardized in people. Finally, this research provides a new perspective on recognizing human expressions with a portable device. Even when the dataset was made with spontaneous and artificial emotions, there were almost no variations between them. We considered that it was not from the EMG sensor errors. However, when the user needs to respond naturally, the system can detect those minor electrical variations.

In future work, the proposed electronic system will be further expanded and optimized to be used in medical treatments with some samples of people who have low facial mobility or facial muscle limitations.

## Figures and Tables

**Table 1:** Muscles involved in the generation of emotions by FACS (accessed on 15 October 2023) [2].

Emotion	Muscular Basis	FACS Name
Happiness	Orbicularis oculi	Cheek raiser
	Zygomaticus major	Lip corner puller
Anger	Depressor glabellae	Brow lowerer
	Depressor supercilii	Upper lid raiser
	Corrugator supercilii	Lid tightener
	Orbicularis oculi	
	Levator palpebrae superioris	
Surprise	Frontalis	Inner brow raiser
	Levator palpebrae superioris	Outer brow raiser
	Masseter	Upper lid raiser
	Temporal	Jaw drop
Fear	Frontalis	Inner brow raiser
	Orbicularis oculi	Outer brow raiser
	Corrugator supercilii	Brow lowerer
	Depresor supercilii	Upper lid raiser
	Levator superioris	Lid tightener
	Risorius	Lip stretcher
	Masseter	Jaw drop
Disgust	Levator labii superioris	Nose wrinkler
	Depresor Anguli oris	Lip corner depressor
	Levator labii Inferioris	Chin raiser
	Mentails	
Sadness	Frontalis	Inner brow raiser
	Depressor Anguli oris	Brow lowerer
	Corrugator supercilii	Lip corner depressor
	Depresor superciliar	

**Table 2:** Lenovo Thinkpad P50s system specifications.

Feature	Description
Processor	Model: Intel Core i7-6500U (9th Gen)
	Speed: 2.5 GHz
	Cache: 4 MB Intel^®^ Smart Cache
	Instruction Set: 64-bit
Memory	Type: DDR3L-1600
	Speed: 1600 MHz
	Capacity: 32 GB
Storage	type: SATA HDD
	Speed: 5400 RPM
	Capacity: 1 TB
GPU	NVIDIA Quadro M500M
	Memory: 2 GB
Operating System	64-bit Windows 10 Professional Edition

**Table 3:** SNR analysis applied smoothing algorithms into the EMG analog signals.

Parameters	Original	FILTERS			
	**Signal**	**Media Mobile**	**Moving Average**	**Savitzky**	**Gaussian**
Mean	145.50	221.04	218.08	220.86	220.86
SD	78.31	93.62	99.28	98.19	89.30
SNR	1.85	2.36	2.19	2.24	2.47

**Table 4:** Proposed multidimensional architecture of the neural network (DP_1_).

Layer (Type)	Output Shape	Number of Parameters
Input (Dense)	(None, 100, 80)	320
Layer 1 (Dense)	(None, 100, 40)	3240
Layer 2 (Dense)	(None, 100, 20)	820
Layer 3 (Flatten)	(None, 2000)	0
Output (Dense)	(None, 6)	12,006

**Table 5:** Proposed one-dimensional architecture of the neural network (DP_2_).

Layer (Type)	Output Shape	Number of Parameters
Input (Dense)	(None, 100)	30,100
Layer 1 (Dense)	(None, 50)	5050
Layer 2 (Dense)	(None, 25)	1275
Output (Dense)	(None, 6)	156

**Table 6:** Evaluation of ML classification models: Precision (Prec.), recall (Rec.), F1-score (F1-sco.), and accuracy (Acc.).

ML Model	Classification Metrics				
	**Prec.**	**Rec.**	**F1-sco.**	**Error**	**Acc.**
SVM	0.9154	0.9166	0.9154	0.1	0.92
kNN	0.8296	0.8166	0.8153	0.25	0.82
Decision tree	0.9384	0.9333	0.9334	0.13	0.93
Naive Bayes	0.9718	0.9666	0.9667	0.06	0.97
DP_1_	1.00	1.00	1.00	0.0	1.0
DP_2_	0.9436	0.9333	0.9319	0.06	0.93

## Data Availability

The data presented in this study are available on request from the corresponding author.
